# Blinatumomab for treating pediatric B-lineage acute lymphoblastic leukemia: A retrospective real-world study

**DOI:** 10.3389/fped.2022.1034373

**Published:** 2022-10-24

**Authors:** Ying Wu, Yanming Li, Jia Fan, Peijing Qi, Wei Lin, Jie Yang, Huiqing Liu, Xiaoling Wang, Huyong Zheng, Tianyou Wang, Ruidong Zhang

**Affiliations:** ^1^ Hematology Center, Beijing Key Laboratory of Pediatric Hematology Oncology, Beijing Children's Hospital, Capital Medical University, National Center for Children's Health, Beijing, China; ^2^ Department of Pharmacy, Beijing Children's Hospital, Capital Medical University, National Center for Children's Health, Beijing, China

**Keywords:** B-cell acute lymphoblastic leukemia, blinatumomab, pediatric, minimal residual disease, real world

## Abstract

**Objectives:**

Blinatumomab was shown to be safe and effective for consolidation therapy in B-cell acute lymphoblastic leukemia (B-ALL). This study aimed to investigate the effectiveness and safety of blinatumomab in pediatric B-ALL patients in a real-world setting.

**Methods:**

This was a retrospective, observational study that included patients who initiated blinatumomab treatment between October 1, 2020 and June 20, 2022. Patients with B-ALL diagnosis, age below 18 years, and at least one blinatumomab treatment cycle were included. Treatment-related toxicities were assessed.

**Result:**

Totally 23 pediatric patients were included in this study, with a median age of 6 years (range, 2 to 11 years). Blinatumomab therapy was applied for MRD-positive (disease ≥0.01%, *n* = 3) or chemotherapy-ineligible (*n* = 20) B-ALL cases. The median follow-up time was 9 months, and all evaluable patients achieved complete molecular remission with undetectable MRD. Four relapsed B-ALL cases proceeded to hematopoietic stem cell transplantation (HSCT) without further bridging therapy, while the others underwent maintenance chemotherapy after blinatumomab treatment. Grade ≥3 febrile neutropenia, white blood cell decrease and seizure were observed in 57%, 48% and 4.3% of patients, respectively. One case discontinued therapy due to neurologic toxicities. Elevated cytokine levels were observed in 4 patients. In all 23 patients, increased *T*-cell and low B-cell counts (<10/μl) were detected during blinatumomab therapy.

**Conclusion:**

These encouraging results suggest blinatumomab in pediatric B-ALL patients with MRD^+^ or chemotherapy-related toxicities is effective and safe in the short run, although long-term follow-up is still needed.

## Introduction

B-lineage acute lymphoblastic leukemia (B-ALL) is a subtype of acute lymphoblastic leukemia, which is the most prevalent cancer among children and adolescents, with a 5-year overall survival (OS) rate currently approaching 90% (1–3). Despite significant improvements in the efficacy of multi-agent chemotherapy regimens, there are still about 15% of subsequent relapse cases and 10% of disease-related deaths, indicating poor survival and low quality of life (4, 5). Moreover, chemotherapy-related side effects occur, causing a major burden on patients and families (6). Thus, better treatment strategies with higher efficacy and lower toxicity are still needed.

In recent years, the emergence of monoclonal antibodies to surface antigens has represented an important advancement in ALL therapy (7, 8). Blinatumomab is a bispecific *T-*cell engager (BiTE) targeted to CD3 and CD19, with promising clinical efficacy as a monotherapy for eradicating persistent MRD in B-ALL (9). In a phase I/II, open-label trial, 27 (39%; 95% CI, 27% to 51%) patients achieved complete remission (CR) within the first two cycles, 14 (52%) of whom achieved complete minimal residual disease response (10). In a prospective, multicenter phase III trial, 108 patients with R/R B-ALL (<18 years) were randomly assigned to the blinatumomab or standard chemotherapy group (11). After a median follow-up of 31 months, overall survival (OS) was higher in the blinatumomab group compared with the chemotherapy group (83% vs. 60%, 95% CI: 0.15–0.72, *p *= 0.003) (11, 12). In another study of 208 B-ALL patients with high- and intermediate-risk first relapse receiving blinatumomab as post-reinduction consolidation prior to HSCT, Brown et al. found two-year OS rates of 71.3% in the blinatumomab group and 58.4% in the chemotherapy group (95% CI, 0.39–0.98; 1-sided *p* = 0.02) (13). The main adverse effects of blinatumomab are cytokine release syndrome and neurotoxicity (11, 12). Based primarily on these results, the US Food and Drug Administration (FDA) and European Medicines Agency (EMA) approved blinatumomab for the treatment of relapsed/refractory (R/R) B-ALL and CR with MRD ≥ 0.01% B-ALL (14). In a real-world study, Beneduce et al. retrospectively assessed the safety and efficacy of blinatumomab in 39 R/R B-ALL cases, of whom most achieved MRD negativity after blinatumomab treatment (15).

Despite these phase III trials, few reports have evaluated the real-world efficacy and safety of blinatumomab in treating relapsed or primary pediatric B-ALL. This report is a retrospective analysis of our single-center experience administering blinatumomab for the treatment of pediatric B-ALL patients.

## Methods

### Study design

This was a retrospective, observational study that included patients who initiated blinatumomab treatment between October 1, 2020 and June 20, 2022 in routine clinical practice at the Hematology Oncology Center of Beijing Children's Hospital, Capital Medical University, which is a unique, high-volume center integrating clinical care, scientific research, teaching, and training (16). Patients with B-ALL diagnosis, age below 18 years, and treatment with blinatumomab were included. This study was approved by the ethics committee of Beijing Children's Hospital ([2022]-E-090-R). The requirement for informed consent was waived by the committee because of the retrospective nature of the current study, which was performed in accordance with the Declaration of Helsinki.

Clinical records were reviewed to extract data, including baseline demographics, ALL-relevant clinical history, frontline therapy, lymphocyte subsets, peripheral cytokine levels and treatment patterns for blinatumomab.

### Outcome measures

This retrospective observational study evaluated complete MRD response, and the incidence and severity of adverse events (AEs). Hematologic and MRD assessments were performed of bone marrow aspirates and core biopsies obtained at screening, at the end of infusion on day 28 of each cycle. MRD was measured based on next-generation sequencing (NGS, threshold 10^−6^/0.0001%). Complete MRD response was defined as no detectable signal for leukemic cells by NGS. Adverse drug reactions were recorded according to the Common Terminology Criteria for Adverse Events (CTCAE) version 5.0 proposed by the National Cancer Institute. Peripheral lymphocyte subsets were evaluated by FACS analyses. Peripheral blood levels of cytokines, including IL-1β, IL-2, IL-4, IL-5, IL-6, IL-8, IL-10, IL-12p70, IL-17, tumor necrosis factor *α* (TNF-α) and interferon gamma *γ* (IFN-*γ*), were assessed by FACS-based analyses with a limit of quantitation of 2.44 pg/ml. Response, relapse, and genetic risk categories were defined according to the National Comprehensive Cancer Network (NCCN) guidelines: Pediatric Acute Lymphoblastic Leukemia (17). All data were collected from electronic medical records by two reviewers to decrease inconsistency.

### Statistical analyses

Demographic and disease characteristics were assessed by descriptive statistics. Categorical variables were expressed as number and percentage (%). Continuous variables were expressed as mean/median and standard deviation (SD). SPSS statistics version 25.0 and GraphPad Prism version 9.0 were used for all statistical analyses.

## Results

### Characteristics of the study population

From October 1, 2020 to June 20, 2022, a total of 23 patients were included in this study. Median age at the initiation of blinatumomab treatment was 6 years (range, 2 to 11 years). Most patients were male (70%). The baseline characteristics of the 23 patients are shown in Table 1. The enrolled patients were assigned to the MRD^+^ or chemotherapy-ineligible group. Of all patients, 20 (87%) with chemotherapy-associated toxicity precluding standard chemotherapy constituted the chemotherapy-ineligible group, while the remaining with MRD >0.01% constituted the MRD^+^ group. Patients in the chemotherapy-ineligible group had consolidation-associated toxicities, including pancreatitis (12/20), anal mucositis (3/20), cerebral venous thrombosis (1/20), septic shock (1/20), infection (1/20), liver damage (1/20), and hyperlipidemia (1/20). Most of these adverse reactions were caused by peg-asparaginase and high-dose methotrexate during consolidation therapy as reported in CCLG previously. Blinatumomab was administered to all chemotherapy-ineligible patients at the time of recovery from overwhelming toxicity.

**Table 1:** Demographic and Baseline Characteristics.

Characteristic	Patients (*n* = 23)
**Age (years), median (range)**	6 (2–11)
**Sex, *n* (%)**	
Male	16 (70)
Female	7 (30)
**Patients with adverse genetic features, *n* (%)**	
MLL rearrangement	1 (4)
BCR-ABL positive	0 (0)
IKZF1 deletion	5 (22)
Philadelphia chromosome-like	3 (13)
TCF3-ZNF384	1 (4)
**Risk stratification**	
Low	1 (4)
Intermediate	10 (43)
High	12 (52)
**Previous relapse**	
0	19 (83)
1	4 (17)
**CR prior to the first cycle, *n* (%)**	23 (100)
MRD positive	3 (13)
MRD negative	20 (87)
**Ineligible for intensive chemotherapy**	20 (87)
Pancreatitis	12 (52)
Mucositis	3 (13)
Cerebral venous thrombosis	1 (4)
Septic shock	1 (4)
Infection	1 (4)
Liver damage	1 (4)
Hyperlipidemia	1 (4)
**CNS involvement**	2 (9)
**Follow-up, months, median (range)**	9 (1–20)
From diagnosis to the first application, months, median (range)	18 (6–49)
**Blinatumomab cycles, *n* (%)**	
1	19 (83)
2	4 (17)

*n*, number; MRD, minimal residual disease.

Eight subjects had high-risk genetic abnormalities, including 1 MLL arrangement, 4 IKZF1 deletion, and 3 Philadelphia chromosome-like cases. Totally 19 (83%) and 4 (17%) patients were treated in the China Children's Leukemia Group (CCLG)-2018-preB-ALL (ChiCTR1800018935) and CCLG-2008 trials, respectively.

A cycle of blinatumomab treatment was 28 days (4 weeks) of continuous intravenous infusion followed by a 14-day (2 weeks) period without medication. Four of all patients received 2 blinatumomab cycles, and 19 underwent one cycle with one stopping blinatumomab therapy on the second day because of serious seizure. Patient 1 received a stepwise dose escalation from 5 to 15 µg/m^2^ per day after reinduction, while other patients received the full dosage (15 µg/m^2^) from day 1. For the prevention of severe cytokine release syndrome (CRS) and neurologic events, patients with MRD^+^ were treated with oral dexamethasone (5 mg/m^2^) for three days during the pretreatment period of blinatumomab.

### Clinical efficacy

**Figure 1:**
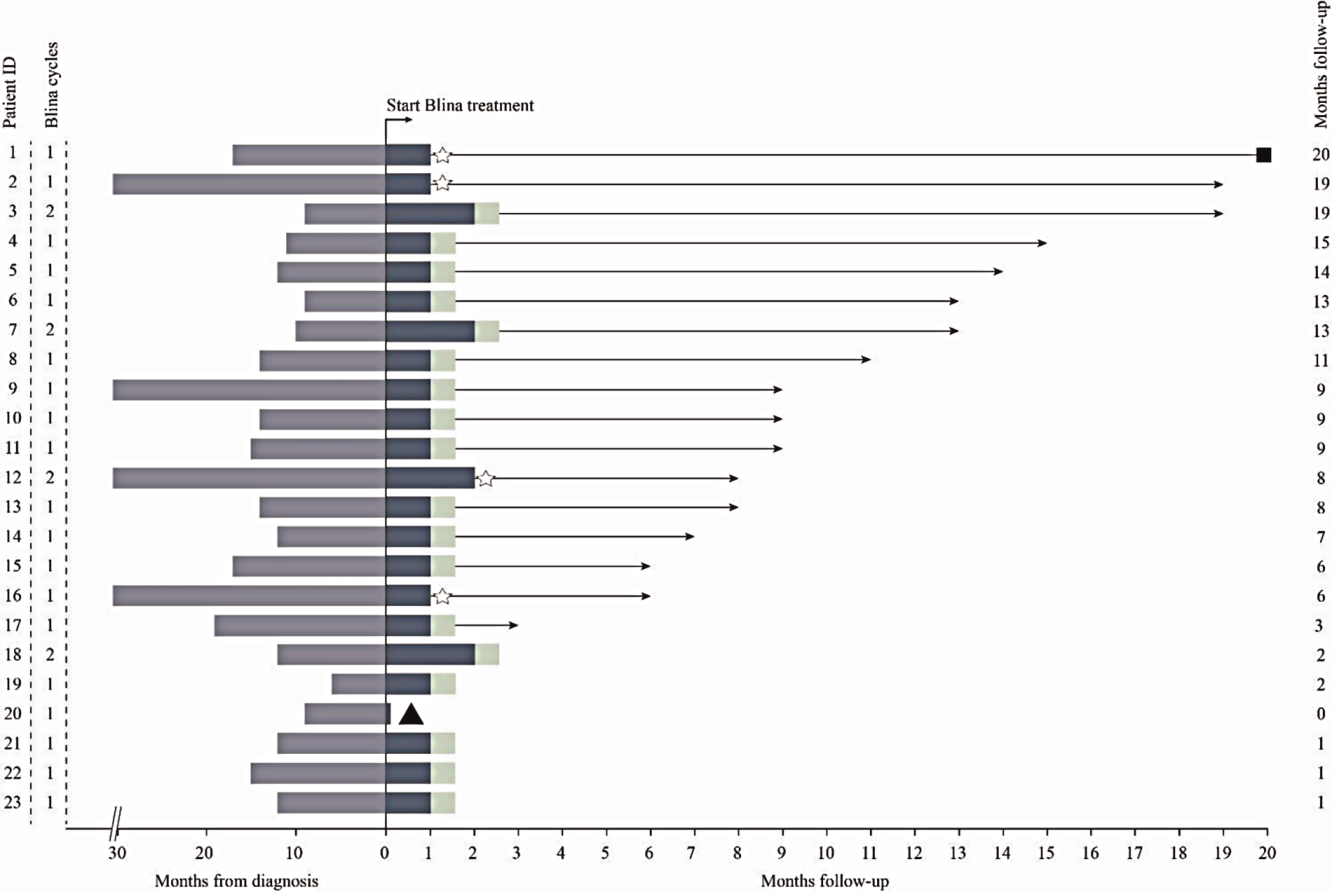
Clinical course of patients treated with blinatumomab (blina). *Blue Shading*, Time From diagnosis; *Light Green Shading*, bridge to maintenance therapy; *Star*, bridge to HSCT; *Triangle*, discontinuation of blinatumomab due to toxicity; *Square*, death.

**Figure 2:**
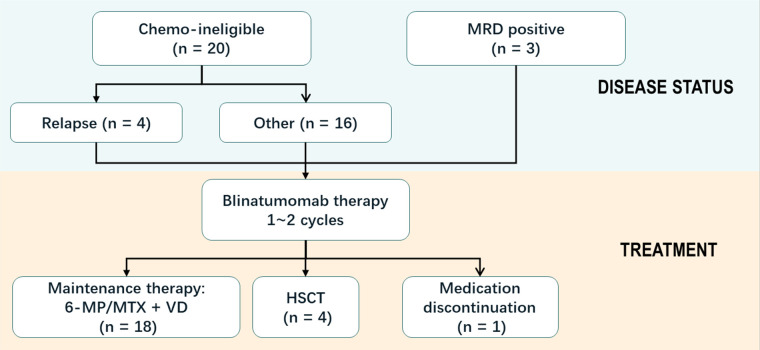
Schema of the therapy of pediatric patients with B-ALL.

Totally 22/23 patients were evaluated for efficacy outcome after blinatumomab therapy. Patient 20 did not complete one course of treatment due to neurotoxicity (Table 2). Hematologic CR had been achieved in all 23 patients prior to the first dose of blinatumomab. NGS-based MRD was detected at 2.33%, 1.04% and 0.28% in patients 2, 12, and 16, respectively, before blinatumomab therapy. The complete MRD response rate in MRD^+^ group was 100%, with 2 (66.7%) patients achieving undetectable MRD during the first cycle and 1 (33.3%) achieving after two cycles. Persistently undetectable MRD after blinatumomab treatment was found in chemotherapy-ineligible patients. Standard maintenance therapy was performed in these individuals following blinatumomab therapy. The mean follow-up time was 9 months, and 34.8% of all patients were followed up for more than 10 months (Figure 1). Four relapsed B-ALL cases proceeded to HSCT after blinatumomab therapy without lymphodepletion. The patients in MRD + group proceeded to maintenance therapy without HSCT due to the fear of their parents and other factors. Totally 20 patients with chemotherapy-associated toxicity recovered and bridged to maintenance therapy in the CCLG-2018-preB-ALL protocol (Figure 2).

**Table 2:** Efficacy outcomes of 23 patients with B-ALL.

Efficacy Outcome	*n*	%
**No. of patients who achieved MRD^-^, *n* (%)**	3 of 3	100
Response in first cycle	2 of 3	67
Response in two cycles	1 of 3	33
**No. of patients proceeding to HSCT**	4 of 23	17
**No. of patients proceeding to maintenance therapy, *n* (%)**	16 of 23	78

*n*, number; MRD, minimal residual disease; HSCT, hematopoietic stem-cell transplantation.

### Safety

Totally 22 out of 23 pediatric patients experienced at least one AE during blinatumomab therapy, regardless of cause. A total of 107 AEs were reported, including 58.9% of grade 1–2 and 42.0% of grade 3–4 (Table 3). The rates of grade 3–4 adverse events included febrile neutropenia (29%), white blood cell decrease (30%), seizure (2%), hepatotoxicity (2%) and fever (2%). None of the patients showed unexpected serious adverse events (SAEs). Sinus tachycardia requiring no medication was developed in eight patients. Five patients developed rash maculo-papular mainly on upper trunk within two weeks of blinatumomab initiation. Two patients showed increased liver enzyme levels (alanine aminotransferase [ALT] and aspartate aminotransferase [AST]) after infusion. Case 22 in the chemotherapy-ineligible group had liver damage, with elevated ALT and AST levels before blinatumomab treatment. Following blinatumomab treatment, glutathione was given as supportive therapy. No subsequent increase in aminotransferase occurred; ALT returned to normal level, and AST returned 1.5-fold to baseline level. Vomiting, diarrhea, abdominal pain, hypercalcemia, PICC-related venous thrombosis, and anal mucositis were observed once, respectively.

**Table 3:** Adverse events according to CTCAE v 5.0.

Toxic Effect	All patients		Chemo-Ineligible Group		MRD positive Group	
	(*n* = 23), *n* (%)		(*n* = 20), *n* (%)		(*n* = 3), *n* (%)	
	Grades 1–2	Grade ≥3	Grades 1–2	Grade ≥3	Grades 1–2	Grade ≥3
**Cytokine release syndrome**	2 (9)	0 (0)	2 (10)	0 (0)	0 (0)	0 (0)
**Neurologic toxicity**						
Seizure	0 (0)	1 (4)	0 (0)	1 (5)	0 (0)	0 (0)
Tremor	1 (4)	0 (0)	1 (5)	0 (0)	0 (0)	0 (0)
**Hematological toxicity**						
Anemia	7 (30)	0 (0)	5 (25)	0 (0)	2 (67)	0 (0)
White blood cell decreased	12 (52)	11 (48)	11 (55)	10 (50)	1 (33)	1 (33)
Neutropenia	2 (10)	15 (65)	1 (5)	13 (65)	1 (33)	2 (67)
Thrombocytopenia	0 (0)	1 (4)	0 (0)	1 (5)	0 (0)	0 (0)
Febrile neutropenia	0 (0)	13 (57)	0 (0)	11 (55)	0 (0)	2 (67)
**Nonhematologic toxicity**						
Fever	15 (65)	3 (13)	12 (60)	3 (15)	3 (100)	0 (0)
Sinus tachycardia	8 (35)	0 (0)	7 (35)	0 (0)	1 (33)	0 (0)
Rash maculo-papular	5 (22)	0 (0)	5 (25)	0 (0)		
Nausea	3 (13)	0 (0)	3 (15)	0 (0)	0 (0)	0 (0)
Vomiting	1 (4)	0 (0)	1 (5)	0 (0)	0 (0)	0 (0)
Diarrhea	1 (4)	0 (0)	1 (5)	0 (0)	0 (0)	0 (0)
Abdominal pain	1 (4)	0 (0)	1 (5)	0 (0)	0 (0)	0 (0)
Increased serum ALT or AST	1 (4)	1 (4)	1 (5)	0 (0)	0 (0)	1 (33)
Hypercalcemia	1 (4)	0 (0)	1 (5)	0 (0)	0 (0)	0 (0)
PICC-venous thrombosis	1 (4)	0 (0)	1 (5)	0 (0)	1 (0)	2 (0)
Anal mucositis	1 (4)	0 (0)	1 (5)	0 (0)	0 (0)	0 (0)
**Discontinuation of blinatumomab because of AE occurrence**	1 (4)		1 (5)	0 (0)	0 (0)	0 (0)

Among the 23 patients, CRS and immune effector cell-associated neurotoxicity syndrome (ICANS) occurred within the first week after administration. CRS was observed in 3 patients (10.5%), with fever and substantial elevations in laboratory parameters such as IL5, IL6, IL10 and IFN-*γ*. The degree of CRS was mild, and the disorder was spontaneously resolved without medication. Two of the patients experienced ICANS, including tremor (grade 1) and seizure (grade 3). Patient 20 discontinued blinatumomab after days in the first cycle due to ICANS.

### Lymphocyte and cytokine levels

**Figure 3:**
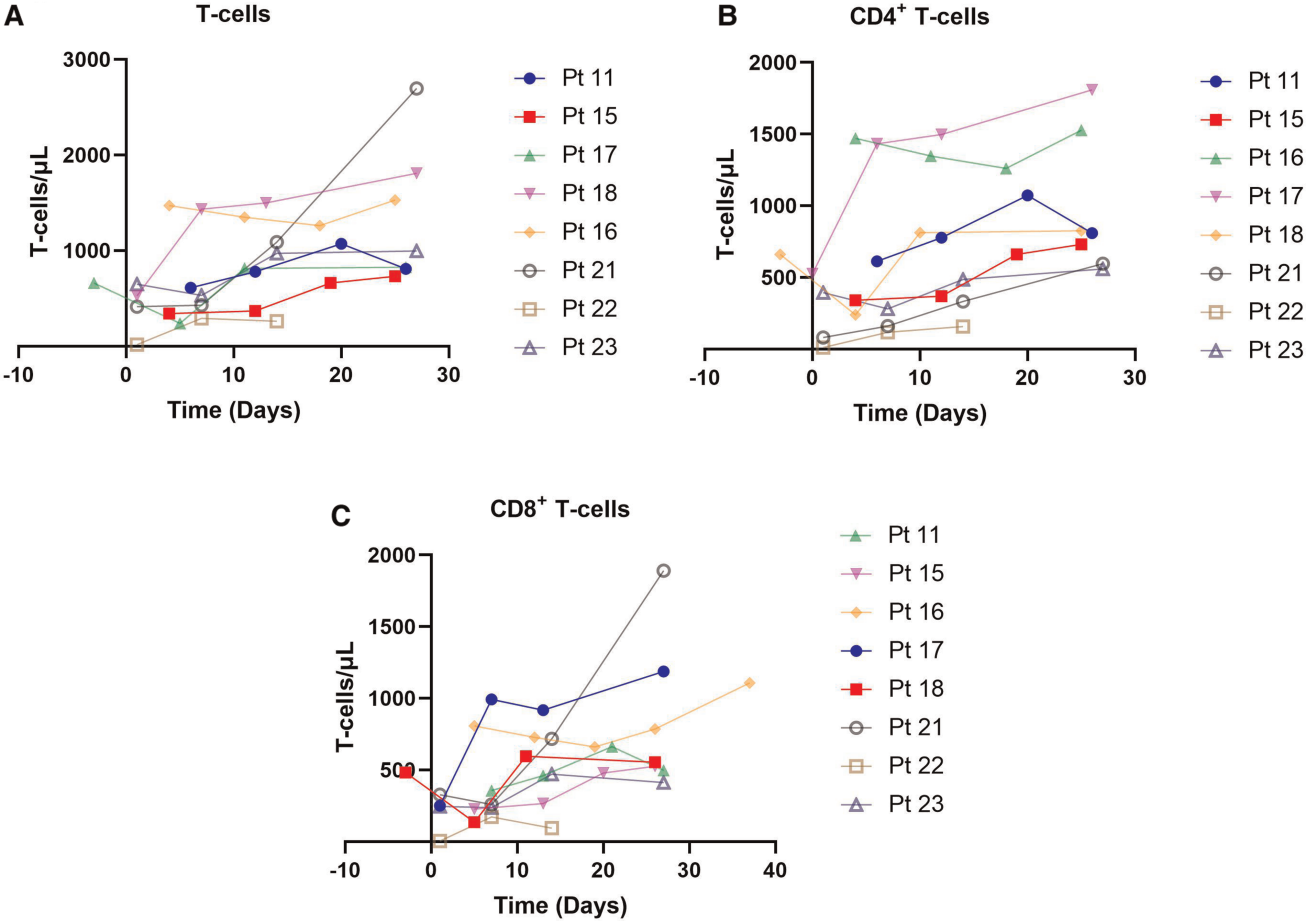
*T*-cells observed in 8 patients. (**A**) *CD8* *+* *T-cells*, chemotherapy-eligible, 7-month follow-up. (**B**) *CD8* *+* *T-cells*, chemotherapy-eligible, 2-month follow-up. (**C**) *CD3* *+* *T-cells*, chemotherapy-eligible, with 1-month follow-up.

**Figure 4:**
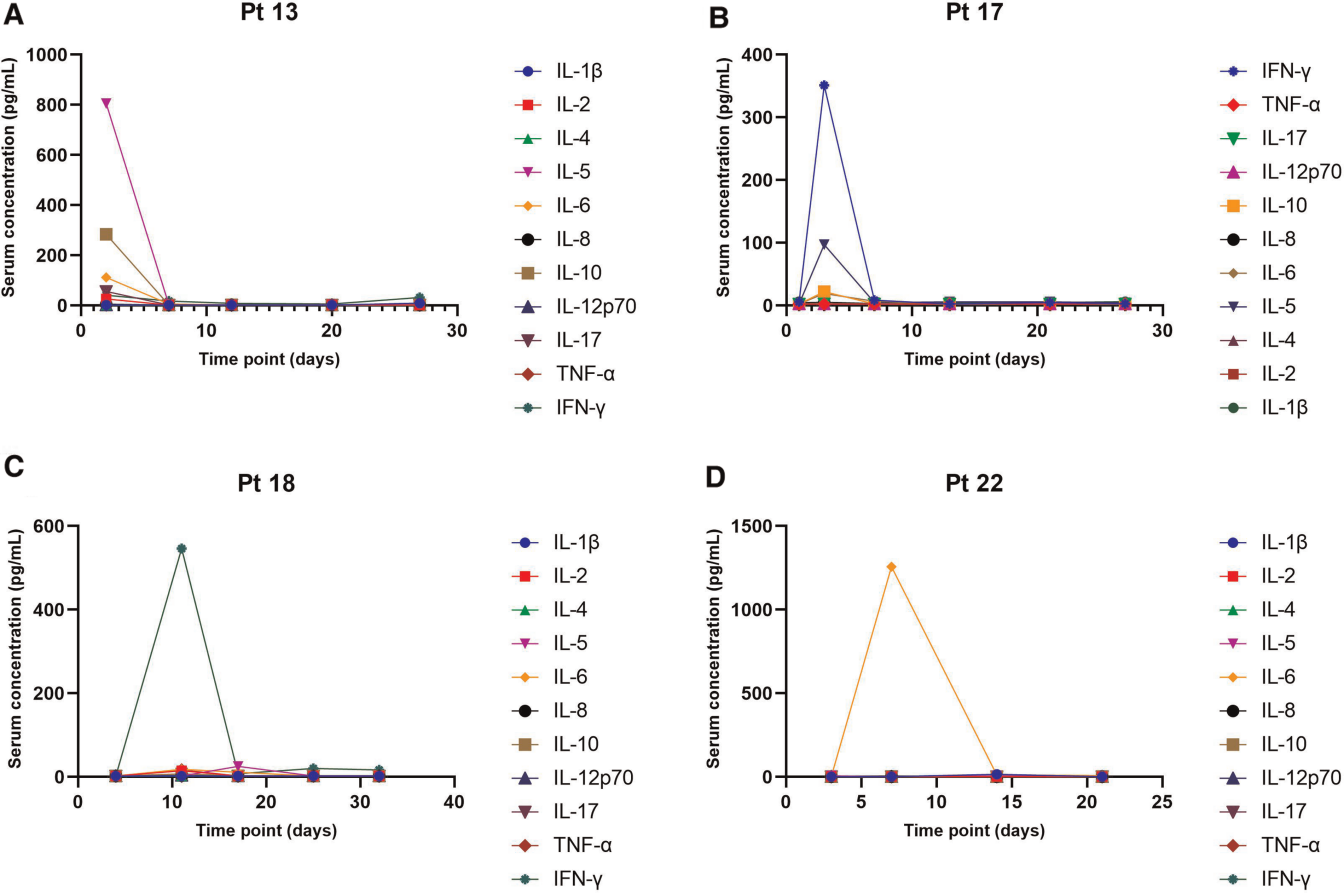
Serum concentrations of cytokines. (**A**) *Patient 13*, chemotherapy-eligible, 7-month follow-up. (**B**) *Patient 17*, chemotherapy-eligible, 2-month follow-up. (**C**) *Patient 18*, chemotherapy-eligible, with 1 month of follow-up. (**D**) *Patient 22*, chemotherapy-eligible, with 1 month of follow-up.

Totally 20 assessable patients in the chemotherapy-ineligible group with low B-cell count (<10/μl) at baseline and after blinatumomab therapy. A slight increase in *T*-cells was observed in peripheral blood samples from 8 patients (Figure 3A). The median count of CD3^+^ T cells was 0.725 × 10^9^ /L at baseline vs. 1.32 × 10^9^ /L at the end of blinatumomab treatment (Supplementary Table S1). In all 8 assessable patients, the absolute CD4^+^ and CD8^+^ T cell counts increased during blinatumomab treatment (Figures 3B,C). Regulatory *T* cells (Tregs) were analyzed in 7 out of the 23 B-ALL patients. The absolute counts of Tregs slightly increased from a median of 7.59 at baseline to 10.06 on Day 27 (*p* = 0.128). The concentrations of cytokines were evaluated in all 20 patients. High levels of cytokines were observed in 4 patients (Figures 3, 4). The maximum cytokine levels were detected for IL-6 (1256.92 pg/ml) on day 7, followed by IL-5 (805.24 pg/ml) on day 1, IFN-*γ* (546.29 pg/ml) on day 11 and IL-10 (283.89 pg/ml) on day 1.

## Discussion

In recent years, blinatumomab has been proven to be safe and feasible in clinical trials after approval by authorities in real-world studies among R/R B-ALL (13, 11, 18). The successful experiences in R/R B-ALL has led to an increasingly use of earlier therapy for B-ALL (19, 10). However, blinatumomab for patients with severe chemotherapy-associated toxicities has not been robustly researched. To our knowledge, there are few real-world studies that have assessed the outcomes of blinatumomab in pediatric patients with intensive chemotherapy-associated toxicity (20, 21). In the present study, we evaluated the safety, efficacy, B and *T* cell responses, and cytokine release of blinatumomab for 23 children with B-ALL based on a large sample size.

In this study, the aim of blinatumomab therapy for B-ALL patients was to supply a “bridge” to HSCT or further therapy, which is required to achieve long-term survival. The outcome here supports findings by previous studies suggesting that such treatment is feasible and safe in pediatric B-lineage ALL with either MRD^+^ or various complications, including sepsis, pancreatitis, oral mucositis and others. Fewer CRS and neurotoxicity occurred, possibly associated with low tumor load (22). Seizure in one patient was recorded during the first day after the start of blinatumomab infusion.

Probably due to prior chemotherapy, all assessable patients in this study had no or barely detectable B cells in peripheral blood at baseline (23). The observation of low B-cell count at the end of treatment suggested the high cytotoxicity of blinatumomab by depleting new B cells (24). Consistent with previous studies, there is an increase in absolute number of *T* cells, which most likely results from CD3-engaging bispecific antibodies. Compared with the findings reported in other articles, *T*-cell activation in this study was much lower (25). This may be related to the low tumor load of the included population, leading to reduced immune activation. However, no depletion of peripheral *T*-cells was observed at the beginning of therapy as previously reported. A possible explanation for this discrepancy may be that we did not detect peripheral lymphocytes at infusion initiation and baseline in the short duration of the present study. Tregs are known to play an important role in the efficacy of immunotherapy (25). However, the slightly elevated Treg level had no statistical significance in the peripheral blood. This may be the basis for further understanding of the immunopharmacological response of blinatumomab.

Some pediatric ALL patients are intolerant to standard chemotherapy and/or have high-risk prognostic factors, and may develop relapsed/refractory acute lymphoblastic leukemia. Blinatumomab treatment, with only temporary and transient myelosuppression, resulted in greater survival benefit than chemotherapy (26). In recent years, reports moved blinatumomab into the front line of the treatment of childhood B-ALL (27). Elitzur et al. (20) reported 11 patients administered blinatumomab who all successfully bridged to further therapy. Daniel et al. (21) presented 15 pediatric ALL cases with invasive fungal disease (IFD) treated with blinatumomab bridging therapy who received ongoing targeted treatment for ALL whilst recovering from IFD. Our study provides further evidence that blinatumomab is well tolerated in patients with severe chemotherapy-related toxicities.

Since this retrospective study was conducted in a single center, some limitations were unavoidable. The first limitation was the small sample size, even though we included all pediatric B-ALL patients administered blinatumomab in this study. Another limitation was the short-term follow-up time, which was only 9 months. Therefore, long-term follow-up is still needed.

## Conclusion

In summary, the current results support the use of the immunotherapeutic drug blinatumomab in the short term among pediatric B-ALL patients with MRD^+^ or chemotherapy-related toxicities, with good efficacy and safety. As the use of blinatumomab expands, further studies are required to ascertain the long-term efficacy and toxicities of blinatumomab, to identify more details about T cell activation triggered by blinatumomab.

## Data Availability

The original contributions presented in the study are included in the article/Supplementary Material, further inquiries can be directed to the corresponding author/s.
