# Corrigendum: Blinatumomab for treating pediatric B-lineage acute lymphoblastic leukemia: A retrospective real-world study

**DOI:** 10.3389/fped.2022.1083730

**Published:** 2022-12-12

**Authors:** Ying Wu, Yanming Li, Jia Fan, Peijing Qi, Wei Lin, Jie Yang, Huiqing Liu, Xiaoling Wang, Huyong Zheng, Tianyou Wang, Ruidong Zhang

**Affiliations:** ^1^ Hematology Center, Beijing Key Laboratory of Pediatric Hematology Oncology, Beijing Children’s Hospital, Capital Medical University, National Center for Children’s Health, Beijing, China; ^2^ Department of Pharmacy, Beijing Children’s Hospital, Capital Medical University, National Center for Children’s Health, Beijing, China

**Keywords:** b-cell acute lymphoblastic leukemia, blinatumomab, pediatric, minimal residual disease, real world

A Corrigendum on Blinatumomab for treating pediatric B-lineage acute lymphoblastic leukemia: A retrospective real-world study By Wu Y, Li Y, Fan J, Qi P, Lin W, Yang J, Liu H, Wang X, Zheng H, Wang T, Zhang R (2022). Front. Pediatr. 10:1034373. doi: 10.3389/fped.2022.1034373

There were errors in the published article. A correction has been made to **Results**, subsection *Characteristics of the study population*, paragraph two. “Eight subjects had high-risk genetic abnormalities, including 1 MLL arrangement, 4 IKZF1 deletion, and 3 Philadelphia chromosome-like cases” has been changed to “Ten subjects had high-risk genetic abnormalities, including 1 MLL rearrangement, 5 IKZF1 deletion, 3 Philadelphia chromosome-like and 1 TCF3-ZNF384 cases.”

A correction has been made to **Results**, subsection *Characteristics of the study population*, paragraph three. “Three of all patients received 2 blinatumomab cycles, and 22 underwent one cycle with one stopping blinatumomab therapy on the second day because of serious seizure” has been changed to “Four of all patients received 2 blinatumomab cycles, and 19 underwent one cycle with one stopping blinatumomab therapy on the second day because of serious seizure.”

A correction has been made to **Results**, subsection *Clinical efficacy*, paragraph one. “Totally 20 patients with chemotherapy-associated toxicity recovered and bridged to maintenance therapy in the CCLG-2018-preB-ALL” has been changed to “15 patients with chemotherapy-associated toxicity recovered and bridged to maintenance therapy in the CCLG-2018-preB-ALL.”

A correction has been made to Results, subsection *Safety*, paragraph one. “A total of 106 AEs were reported, including 58.5% of grade 1–2 and 41.5% of grade 3–4” has been changed to “A total of 108 AEs were reported, including 58.3% of grade 1–2 and 41.6% of grade 3–4.”

A correction has been made to **Results**, subsection *Safety*, paragraph one. “The rates of grade 3–4 adverse events included febrile neutropenia (29%), white blood cell decrease (30%), seizure (2%), hepatotoxicity (2%) and fever (2%)” has been changed to “The rates of grade 3–4 adverse events included febrile neutropenia (57%), white blood cell decrease (48%), thrombocytopenia (4%), seizure (4%), hepatotoxicity (4%) and fever (13%).”

A correction has been made to **Results**, subsection *Lymphocyte and cytokine levels*. “The median count of CD3^+^ T cells was 1.148 × 10^9^ /l at baseline vs. 1.7 × 10^9^ /l at the end of blinatumomab treatment (Supplementary Table S1)” has been changed to “The median count of CD3^+^ T cells was 0.725 × 10^9^ /L at baseline vs. 1.32 × 10^9^ /L at the end of blinatumomab treatment (Supplementary Table S1)”.

A correction has been made to **Results**, subsection *Lymphocyte and cytokine levels*. “The absolute counts of Tregs slightly increased from a median of 9.46 at baseline to 11.31 on Day 27 (*p* = 0.47)” has been changed to “The absolute counts of Tregs slightly increased from a median of 7.59 at baseline to 10.06 on Day 27 (*p* = 0.128).”

A correction has been made to **Results**, subsection *Safety*, paragraph two. “CRS was observed in 3 patients (10.5%)” has been changed to “CRS was observed in 3 patients (13.0%).”

A correction has been made to **Results**, subsection *Lymphocyte and cytokine levels,* paragraph one. “High levels of cytokines were observed in 3 patients (Figures 3, 4)” has been changed to “High levels of cytokines were observed in 4 patients (Figure 4).”

A correction has been made to **Discussion**. “we evaluated the safety, efficacy, B and *T* cell responses, and cytokine release of blinatumomab for 20 children with B-ALL based on a large sample size” has been changed to “we evaluated the safety, efficacy, B and *T* cell responses, and cytokine release of blinatumomab for 23 children with B-ALL based on a large sample size.”

**Error in the Ethics statement**

Furthermore, a change was made to the **Ethics** statement: “The studies involving human participants were reviewed and approved by This study was approved by the Institutional Review Boards of Beijing Children's Hospital ([2022]-E-090-R)” has been changed to “The study involving human participants was reviewed and approved by the Institutional Review Boards of Beijing Children's Hospital ([2022]-E-090-R).”

**Error in Tables**

In the published article, there was an error in Table 2. The number of patients proceeding to maintenance therapy should have been written as 18 instead of 16. The corrected Table 2 and its caption appear in the following.

Finally, in Table 3, the number and percentage of Grades 1–2 cytokine release syndrome in all patients, Grades 1–2 cytokine release syndrome in the Chemo-ineligible group, and PICC-venous thrombosis in the MRD-positive group were incorrect and the number and percentage of Rash maculopapular in the MRD-positive Group were missed. The corrected Table 3 and its caption appear in the following.

The authors apologize for these errors and state that these do not change the scientific conclusions of the article in any way. The original article has been updated.

**Table 2:** Efficacy outcomes of 23 patients with B-ALL.

Efficacy outcome	*n*	%
No. of patients who achieved MRD	3 of 3	100
Response in the first cycle	2 of 3	67
Response in two cycles	1 of 3	33
No. of patients proceeding to HSCT	4 of 23	17
No. of patients proceeding to maintenance therapy	18 of 23	78

*n*, number; MRD, minimal residual disease; HSCT, hematopoietic stem-cell transplantation.

**Table 3:** Adverse events according to CTCAE v 5.0.

Toxic effect	All patients (*n* = 23), *n* (%)		Chemo-ineligible group (*n* = 20), *n* (%)		MRD-positive group (*n* = 3), *n* (%)	
	Grades 1–2	Grade ≥3	Grades 1–2	Grade ≥3	Grades 1–2	Grade ≥3
Cytokine release syndrome	3 (13)	0 (0)	3 (15)	0 (0)	0 (0)	0 (0)
*Neurologic toxicity*						
Seizure	0 (0)	1 (4)	0 (0)	1 (5)	0 (0)	0 (0)
Tremor	1 (4)	0 (0)	1 (5)	0 (0)	0 (0)	0 (0)
*Hematological toxicity*						
Anemia	7 (30)	0 (0)	5 (25)	0 (0)	2 (67)	0 (0)
White blood cells decreased	12 (52)	11 (48)	11 (55)	10 (50)	1 (33)	1 (33)
Neutropenia	2 (10)	15 (65)	1 (5)	13 (65)	1 (33)	2 (67)
Thrombocytopenia	0 (0)	1 (4)	0 (0)	1 (5)	0 (0)	0 (0)
Febrile neutropenia	0 (0)	13 (57)	0 (0)	11 (55)	0 (0)	2 (67)
*Nonhematologic toxicity*						
Fever	15 (65)	3 (13)	12 (60)	3 (15)	3 (100)	0 (0)
Sinus tachycardia	8 (35)	0 (0)	7 (35)	0 (0)	1 (33)	0 (0)
Rash maculopapular	5 (22)	0 (0)	5 (25)	0 (0)	0 (0)	0 (0)
Nausea	3 (13)	0 (0)	3 (15)	0 (0)	0 (0)	0 (0)
Vomiting	1 (4)	0 (0)	1 (5)	0 (0)	0 (0)	0 (0)
Diarrhea	1 (4)	0 (0)	1 (5)	0 (0)	0 (0)	0 (0)
Abdominal pain	1 (4)	0 (0)	1 (5)	0 (0)	0 (0)	0 (0)
Increased serum ALT or AST	1 (4)	1 (4)	1 (5)	0 (0)	0 (0)	1 (33)
Hypercalcemia	1 (4)	0 (0)	1 (5)	0 (0)	0 (0)	0 (0)
PICC-venous thrombosis	1 (4)	0 (0)	1 (5)	0 (0)	0 (0)	0 (0)
Anal mucositis	1 (4)	0 (0)	1 (5)	0 (0)	0 (0)	0 (0)
Discontinuation of blinatumomab because of AE occurrence	1 (4)		1 (5)	0 (0)	0 (0)	0 (0)

