# Supplementary material for: Blinatumomab for treating pediatric B-lineage acute lymphoblastic leukemia: A retrospective real-world study
Source: Front Pediatr. 2022 Oct 24;10:1034373. doi: 10.3389/fped.2022.1034373 (PMC9638881; doi:10.3389/fped.2022.1034373)
Supplement: Supplementary file 1 [file Datasheet1.docx]

**Supplemental materials**

**Supplemental Table 1. Peripheral CD3^+^ T-cells evaluated in 8 patients**

| **Patient ID** | **D 0 (10^9^/L)** | **D 27 (10^9^/L)** |
| --- | --- | --- |
| **4** | 1.2 | 1.64 |
| **5** | 0.63 | 1.34 |
| **6** | 1.03 | 1.19 |
| **7** | 0.56 | 0.43 |
| **8** | 1.5 | 1.72 |
| **18** | 0.82 | 2.18 |
| **21** | 0.41 | 2.70 |
| **23** | 0.65 | 1.00 |

**Supplemental Table 2. Treg T-cells (%) in peripheral blood**

| **Time (Day)** | **Pt 4** | **Pt 5** | **Pt 6** | **Pt 7** | **Pt 8** | **Pt 9** | **Pt 11** |
| --- | --- | --- | --- | --- | --- | --- | --- |
| **D 0** | 6.71 | 7.52 | 8.65 | 7.59 | 8.96 | 19.73 | 7.08 |
| **D 27** | 8.7 | 10.06 | 12.56 | 9.57 | 6.73 | 21.18 | 10.38 |

**Supplemental Table 3. CD3^+^ T-cells, CD4^+^ T-cells, and CD8^+^ T-cells in peripheral blood**

| **PtID** | **Time (Days)** | **CD3+ T-cells/μL** | **CD4+ T-cells/μL** | **CD8+ T-cells/μL** | **CD4+, CD27+,CD45RA+** | **CD8+, CD27+,CD45RA+** | **CD4+, CD27-,CD45RA+** | **CD8+, CD27-,CD45RA+** | **CD4+, CD27-,CD45RA-** | **CD8+, CD27-,CD45RA-** | **CD4+, CD27+,CD45RA-** | **CD8+, CD27+,CD45RA-** |
| --- | --- | --- | --- | --- | --- | --- | --- | --- | --- | --- | --- | --- |
| **11** | **6** | **612** | **195** | **355** | **-** | **-** | **-** | **-** | **-** | **-** | **-** | **-** |
|  | **12** | **778** | **240** | **460** | **-** | **-** | **-** | **-** | **-** | **-** | **-** | **-** |
|  | **20** | **1072** | **278** | **662** | **-** | **-** | **-** | **-** | **-** | **-** | **-** | **-** |
|  | **26** | **809** | **222** | **498** | **-** | **-** | **-** | **-** | **-** | **-** | **-** | **-** |
| **15** | **4** | **340** | **102** | **228** | **26** | **27** | **1** | **26** | **16** | **116** | **59** | **58** |
|  | **12** | **369** | **112** | **266** | **25** | **38** | **0** | **32** | **21** | **94** | **66** | **102** |
|  | **19** | **660** | **162** | **479** | **33** | **59** | **1** | **65** | **28** | **159** | **101** | **196** |
|  | **25** | **730** | **172** | **526** | **29** | **86** | **0** | **100** | **37** | **143** | **105** | **197** |
| **16** | **4** | **1471** | **537** | **808** | **292** | **365** | **0** | **50** | **37** | **191** | **208** | **201** |
|  | **11** | **1348** | **515** | **728** | **201** | **307** | **0** | **73** | **43** | **183** | **271** | **166** |
|  | **18** | **1261** | **516** | **660** | **238** | **354** | **1** | **66** | **34** | **119** | **243** | **120** |
|  | **25** | **1528** | **523** | **786** | **268** | **387** | **0** | **62** | **46** | **188** | **209** | **149** |
| **17** | **0** | **525** | **208** | **250** | **64** | **203** | **0** | **2** | **17** | **3** | **127** | **42** |
|  | **6** | **1433** | **300** | **992** | **132** | **769** | **0** | **6** | **18** | **6** | **149** | **211** |
|  | **12** | **1498** | **424** | **918** | **153** | **709** | **5** | **17** | **33** | **6** | **233** | **185** |
|  | **26** | **1809** | **556** | **1188** | **216** | **1004** | **0** | **4** | **39** | **7** | **301** | **172** |
| **18** | **-3** | **660** | **214** | **482** | **36** | **85** | **1** | **4** | **19** | **26** | **158** | **368** |
|  | **4** | **239** | **111** | **136** | **24** | **17** | **0** | **13** | **9** | **19** | **78** | **87** |
|  | **10** | **813** | **268** | **595** | **34** | **81** | **1** | **9** | **23** | **42** | **211** | **464** |
|  | **25** | **826** | **343** | **554** | **44** | **131** | **0** | **11** | **22** | **42** | **277** | **371** |
